# Validation of 2 urine pH measuring techniques in a prepartum negative dietary cation-anion difference diet and the relationship with production performance

**DOI:** 10.3168/jdsc.2021-0130

**Published:** 2021-11-18

**Authors:** L.K. Fehlberg, A. Pineda, F.C. Cardoso

**Affiliations:** 1 Department of Animal Sciences, University of Illinois, Urbana 61801; 2 Department of Animal Biosciences, University of Guelph, Guelph, ON, Canada, N1G 2W1

## Abstract

Negative dietary cation-anion difference (DCAD) diets have been implemented to combat hypocalcemia, a common peripartal disease in dairy cows; however, the extent of compensatory metabolic acidosis necessary and the subsequent effects on performance are still debated. Additionally, there is a need for an inexpensive, accurate method to measure urine pH on farm during the prepartum period to assess the extent of metabolic acidosis achieved by negative DCAD diets. Therefore, this experiment was conducted to determine the accuracy of Fisher pH sticks (pHF; ThermoFisher Scientific) and pHion balance test strips (pHI; pHion Balance) compared with a portable pH meter (pHP; Accumet AP115, ThermoFisher Scientific) in measuring urine pH (UpH) and the effect of UpH on pre- and postpartum dry matter intake (DMI), milk, and milk composition yields. Cows consumed a total mixed ration with a DCAD of −118 mEq/kg for 4 wk prepartum and 397 mEq/kg for 4 wk postpartum. Prepartum UpH measurements (n = 75) for each cow were averaged and used to classify cows in terms of urine pH as low (UpH ≤5.54; mean ± standard deviation; 5.44 ± 0.07), medium (UpH >5.54 and ≤5.90; 5.67 ± 0.09), or high (UpH >5.90; 6.42 ± 0.36). Cows were milked twice a day, and milk samples were taken on d 7 ± 1.3, 14 ± 1.4, and 28 ± 1.1 relative to calving. Milk yield and DMI were recorded daily and averaged weekly. Bland-Altman plots and Lin's concordance correlation coefficient (CCC) were used to assess the agreement between pHP and pHF or pHI (n = 375). Receiver operating characteristic curves were used to determine the threshold with pHF and pHI that best discriminated between UpH >5.75 and ≤5.75 compared with pHP, and area under the curve (AUC) was used to assess the accuracy. At the UpH threshold of 5.75 for pHF and pHI, the sensitivity, specificity, and AUC were 89.5 and 87.4, 99.1 and 97.0, and 0.94 and 0.92, respectively. The CCC was 0.93 for pHF and pHI, indicating near-perfect agreement with pHP. The UpH did not affect pre- or postpartum DMI. There was a tendency for a UpH × week interaction for milk yield, in which milk yields were less for cows in the low and medium groups. In conclusion, pHI and pHF are accurate measurements for UpH, and UpH did not affect DMI; however, when UpH was low or medium, milk yield was decreased at wk 1 postpartum.

## Highlights

• Accurate and cheap measurement of urine pH is desirable for the dairy industry.
• The two urine pH strips tested were accurate in measuring urine pH in prepartum cows.
• Varying urine pH was not associated with altered dry matter intake prepartum.

## Graphical Abstract

**Figure:**
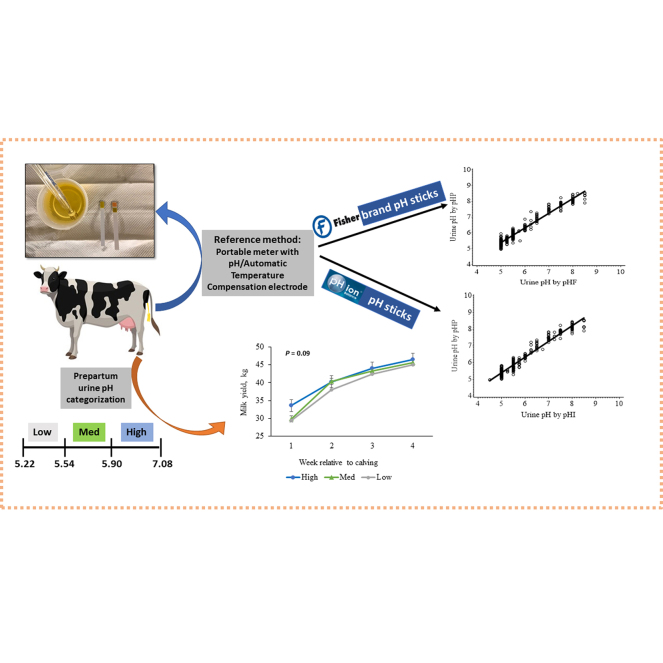
**Summary:** Acidified diets prepartum are utilized in dairy cows to decrease the likelihood of hypocalcemia; however, the effect of the extent of acidification on cow performance is still debated. Our objective was to validate the accuracy of 2 pH strips to measure urine pH (categorized as low, medium, or high) in dairy cows consuming an acidified diet prepartum and the association of urine pH with production performance. We determined that both pH strips are an accurate and affordable method to determine urine pH. Additionally, varying urine pH was not associated with dry matter intake when cows consumed an acidified diet; however, milk yield was moderately affected during week 1 postpartum when average urine pH prepartum was <5.67.

In the dairy industry, there is an ever-present concern about health during the transition from gestation to lactation due to the high prevalence of metabolic disorders. Clinical and subclinical hypocalcemia contributes substantially to stress during the transition period for both cow and manager, potentially costing up to $246/case (Liang et al., 2017). Hypocalcemia occurs when the requirement for Ca increases drastically around calving to provide calcium for colostrum and milk production (Charbonneau et al., 2006). Because Ca homeostasis is tightly regulated in the cow, improper adaptation to lactation can lead to decreased blood calcium, resulting in hypocalcemia (Goff and Horst, 1997). Alleviation methods have been recommended to increase Ca mobilization from the bones before calving to prepare the cow for the increased Ca demand after calving. Historically, decreasing dietary Ca below requirements prepartum accompanied by a positive DCAD has been suggested; however, Ca requirements can be as low as 25 g/d per cow, which is very difficult to achieve in common TMR diets (Crenshaw et al., 2011). Alternatively, an acidified diet achieved through negative DCAD can be used prepartum to induce a compensatory metabolic acidosis (Goff et al., 2014). Metabolic acidosis causes increased Ca excretion in the urine and increased bone resorption and sensitivity of tissues to hormonal regulation to maintain Ca homeostasis (Rodríguez et al., 2016). This allows the cow to adjust to a greater demand for calcium before calving, allowing for faster adaptation after calving.

In addition to increasing excretion of Ca, a negative DCAD will also decrease urine pH (**UpH**), which typically reflects blood pH. For this reason, UpH can be used to determine the degree of metabolic acidosis a cow is experiencing (Goff and Koszewski, 2018). During the metabolic alkalosis state in dairy cows, UpH is typically ≥8.0 (Goff and Koszewski, 2018). However, the degree of acidification and associated UpH prepartum that are ideal for health and production postpartum are still debated (Cardoso et al., 2020). Previously, studies noted UpH values below 7.0, with most cows near 6.0, when negative DCAD diets were consumed (Jardon, 1995; Charbonneau et al., 2006), deeming the diets partially acidified (Cardoso et al., 2020). However, a fully acidified diet with a target UpH of 5.5 to 6.0 has recently received attention, specifically to allow for increased Ca in the diet prepartum while maintaining metabolic acidosis due to the cation properties of Ca (Cardoso et al., 2020; Glosson et al., 2020). A fully acidified diet allowed for the addition of Ca at 2% of DM to the prepartum diet without compromising metabolic acidosis, resulting in improved health and reproductive success postpartum (Glosson et al., 2020; Ryan et al., 2020). To achieve the target degree of acidification on farm, accurate and inexpensive measurements of UpH are needed. Previously, the gold standard to measure pH was a portable pH meter; however, glass electrode pH meters require frequent calibration and training to ensure accuracy, thereby decreasing the popularity of this method (Constable et al., 2019). Therefore, a cheaper alternative is desired that allows for rapid determination of UpH on farm but maintains the accuracy of the gold standard.

All experimental procedures were approved by the University of Illinois (Urbana-Champaign) Institutional Animal Care and Use Committee (#18157) and were a part of a larger study (Fehlberg et al., 2020). A detailed description of this study is provided in Fehlberg et al. (2020). Briefly, the experimental period was from September 2018 to June 2019. A total of 83 multiparous pregnant Holstein cows with BW (mean ± SD) = 791 ± 84 kg were blocked by parity (3.3 ± 1.1), previous 305-d mature-equivalent milk production (11,363 ± 1,860 kg), expected calving date, and BCS during the far-off dry period (3.76 ± 0.84).

Cows were dried off at −57 ± 21 d relative to expected calving and consumed a common straw-based diet until −30 d relative to expected calving. Cows were then moved to an enclosed ventilated barn with access to sand-bedded freestalls at −30 d relative to expected calving, where they were fed once daily at approximately 0530 h using an individual feeding system (American Calan Inc.), beginning the experimental period. Diets (TMR) were formulated using AMTS.Cattle.Pro version 4.7 (2017, AMTS LLC) to meet or exceed recommendations. The dry cow diet was formulated for cows at 694 kg of BW, a predicted DMI of 13 kg/d, and to achieve a DCAD of −118 mEq/kg, where DCAD = ([Na^+^] + [K^+^]) – ([Cl^–^] + [S^2–^]). The mean chemical composition (n = 10) of the prepartum TMR (DM basis) was 14.2 ± 0.68% CP, 28.4 ± 2.80% ADF, 44.8 ± 2.75% NDF, 14.0 ± 1.69% starch, and 1.44 ± 0.03 NE_L_ (Mcal/kg of DM). The DCAD was obtained by dietary ingredients and the addition of an anionic mineral supplement (Animate; Phibro Animal Health Corp.) included at 3.85% of DM. After calving, cows were housed in a tiestall barn until 28 d relative to calving and consumed a postpartum diet formulated for cows at 14 DIM, 733 kg of BW, producing 39 kg of milk/d with a target of 3.7% milk fat and 3.2% milk protein, a predicted DMI of 19 kg/d, and to achieve a positive DCAD of 397 mEq/kg. The mean chemical composition (n = 10) of the postpartum TMR (DM basis) was 16.8 ± 1.06% CP, 20.9 ± 1.77% ADF, 31.3 ± 3.29% NDF, 24.4 ± 2.62% starch, and 1.67 ± 0.05 NE_L_ (Mcal/kg of DM).

Urine was collected once weekly during the experimental period at 0500 h, immediately before feeding. Time points of urine collection were characterized as weeks before calving and included wk −1 (n = 83), −2 (n = 83), −3 (n = 80), and −4 (n = 53). Due to variation in actual calving day, not all cows had urine collected for wk −3 or −4. The number of cows per treatment was calculated to detect a minimum of 7% difference in postpartum DMI between groups, assuming a power of 0.9 and a 2-tailed α of 0.05 (Fehlberg et al., 2020). Cows were manually stimulated until urination, and approximately 80 mL of voided urine was collected via free-catch into a 100-mL urine collection tube (Fisher Scientific) during mid-stream of urination. The tube was immediately closed to decrease exposure to CO_2_ and subsequent increased pH (Bender and Staufenbiel, 2003). Urine pH was measured within 5 min of collection by a single operator using a portable pH meter (**pHP**; Accumet AP115, ThermoFisher Scientific) with a pH/automatic temperature compensation glass electrode (which was considered the gold standard), Fisherbrand pH sticks (**pHF**; ThermoFisher Scientific), and pHion balance test strips (**pHI**; pHion Balance). For this study, both pHF and pHI could detect a minimum pH of 5.0; the pHF scale increased by intervals of 0.5 until 9.0, whereas the pHI scale increased by 0.5 until a pH of 5.5 and then by 0.25 to a pH of 9.0. Fisherbrand pH sticks and pHI were selected due to their ability to measure urine pH as low as 5.0 and for both sticks to have consistent intervals of measurement.

Daily DMI was determined for each cow by weighing refusals and total amounts fed and determining the difference on a DM basis. Cows were fed for 10% refusals to allow for ad libitum feed intake. All cows had free access to water. Milking procedures were explained in detail elsewhere (Fehlberg et al., 2020). Briefly, cows were milked 2 × per day and weights were recorded at each milking. Milk samples were collected at both a.m. and p.m. milkings at (mean ± SD) 7d ± 1.3, 14 ± 1.4, and 28 ± 1.1 relative to calving, composited in proportion to milk yield at each milking, and then shipped to a commercial laboratory (Dairy One Cooperative Inc., Ithaca, NY) to be analyzed for contents of fat, true protein, casein, lactose, SCC, total solids, and MUN using mid-infrared procedures (AOAC International, 1995).

Statistical analyses were performed using the MIXED, REG, and LOGISTIC procedures of SAS (version 9.4, SAS Institute Inc.). Urine pH measured by pHP was divided into terciles and classified for wk −4 as low (UpH ≤5.55; mean ± SD; 5.36 ± 0.15), medium (UpH >5.55 and ≤5.78; 5.65 ± 0.06), or high (UpH >5.78; 6.51 ± 0.57); for wk −3 as low (UpH ≤5.49; 5.35 ± 0.11), medium (UpH >5.49 and ≤5.77; 5.62 ± 0.09), or high (UpH >5.77; 6.62 ± 0.69); for wk −2 as low (UpH ≤5.37; 5.25 ± 0.10), medium (UpH >5.37 and ≤5.65; 5.50 ± 0.08), or high (UpH >5.65; 6.46 ± 0.68); and for wk −1 as low (UpH ≤5.48; 5.36 ± 0.11), medium (UpH >5.48 and ≤5.80; 5.66 ± 0.09), or high (UpH >5.66; 6.48 ± 0.54). Daily prepartum DMI was condensed into weekly averages and analyzed independently each week, using the corresponding low, medium, and high classifications for each week. Prepartum UpH measurements for each cow (n = 75) were then averaged and used to classify cows as low (UpH ≤5.54; mean ± SD; 5.44 ± 0.07), medium (UpH >5.54 and ≤5.90; 5.67 ± 0.09), or high (UpH >5.90; 6.42 ± 0.36) and used for postpartum DMI, and milk, ECM, 3.5% FCM, and milk composition yields. Postpartum DMI and milk yields were condensed to weekly averages. The model included the fixed effects of UpH, week, and their interaction. Cow was the experimental unit and considered a random effect. Week was specified as repeated with cow as subject when analyzing variables measured over time. Denominator degrees of freedom was estimated using the Kenward-Roger method (Littell, 2002). Distribution of the residuals was evaluated to determine normality and homoscedasticity.

Regression and correlation analyses were carried out to estimate the association between UpH determined with pHP and pHF or pHI (n = 375). Correlation coefficients measured the strength of the relationship between pHP and pHF or pHI, not the agreement among them (Bland and Altman, 1986). Consequently, assessing diagnostic test performance with correlation coefficients only may be inappropriate. Therefore, Bland-Altman plots (Bland and Altman, 1986) and Lin's concordance correlation coefficient (**CCC**; Crawford et al., 2007) were used to visualize and quantify, respectively, the agreement between the results from pHP and pHF or pHI. The Durbin-Watson coefficient was used to test for autocorrelation within residuals to determine independence among samples, with a score near 2 indicating zero autocorrelation.

Contingency 2 × 2 tables were created to obtain true-negative, true-positive, false-negative, and false-positive values. These values were used to compute the test characteristics (sensitivity, specificity, and positive and negative predictive values). Sensitivity (**Se**) was calculated as the proportion of urine samples with pH ≤5.75 correctly determined by pHF or pHI. Specificity (**Sp**) was calculated as the proportion of urine samples with pH >5.75 correctly determined by pHF or pHI. Positive predictive value was calculated as the proportion of the urine samples with pH ≤5.75 that were correctly analyzed. Negative predictive value was calculated as the proportion of the urine samples with pH >5.75 that were correctly analyzed. Receiver operating characteristic (**ROC**) curves were constructed to identify the threshold with pHF and pHI that best discriminated between urine samples with pH >5.75 and those ≤5.75 based on the gold standard test. The area under the ROC curve (**AUC**) was used to assess the accuracy of the pHF and pHI thresholds. Statistical significance for all analyses was declared at *P* ≤ 0.05 and trends at 0.05 < *P* ≤ 0.10.

There was excellent correlation between pHF and pHI and the gold standard pHP (r = 0.98; *P* < 0.0001; Figure 1). To determine optimal thresholds and Se and Sp values at varying thresholds, ROC curves were used. When the threshold was set at 5.75, Se was 89.5% (95% CI: 0.85–0.95) and 87.4% (95% CI: 0.82–0.93) and Sp was 99.1% (95% CI: 0.98–1.00) and 97.0% (95% CI: 0.95–0.99) for pHF and pHI, respectively. These values coincided with the greatest AUC used to determine the accuracy of the thresholds. When the AUC is 0.5, discrimination does not exist, whereas when AUC is 1.0, perfect discrimination exists, resulting in a true-positive rate of 1.0 at all false-positive rate values (Swets, 1988). The greatest AUC for pHF and pHI at the threshold of 5.75 was 0.94 (95% CI: 0.91–0.97) and 0.92 (95% CI: 0.89–0.95), respectively. Therefore, when the threshold is set at 5.75, the AUC is considered excellent (AUC >0.90; Swets, 1988) for pHF and pHI. This agrees with a previous study in which a urine dipstick (Multistix-10-SG; Siemens) and pH paper (Hydrion; MicroEssential Laboratory) had excellent agreement, with AUC = 0.991 and 0.995, respectively, compared with a pH meter (Constable et al., 2019), although AUC was slightly less in the current study.

**Figure 1:**
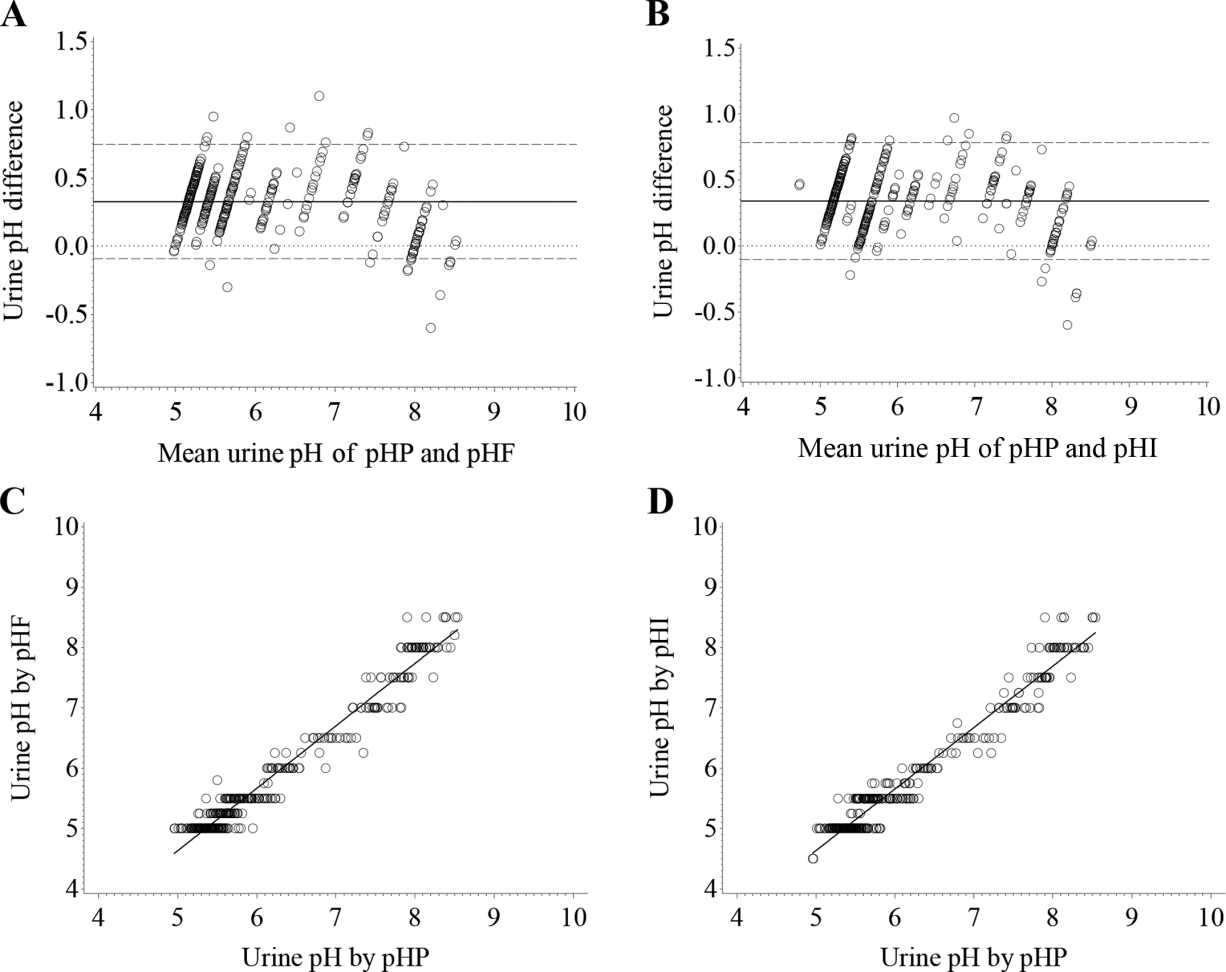
Bland-Altman plot of differences between urine pH (n = 375) determined by a portable pH meter (pHP; Accumet AP115, ThermoFisher Scientific) and that determined using (A) Fisher pH sticks (pHF; ThermoFisher Scientific) and (B) pHion balance test strips (pHI; pHion Balance) plotted against their mean concentrations. The solid line in the middle represents the mean (bias), the upper and lower dashed lines represent the limit of the agreement (bias ± 1.96 SD), and the dotted line indicates bias = 0. Relationship between urine pH determined by pHP and (C) pHF or (D) pHI. (C) Adjusted R^2^ = 0.96, r = 0.98, *P* < 0.0001, *y* = −0.54619 + 1.03552*x*; (D) adjusted R^2^ = 0.96, r = 0.98, *P* < 0.0001, *y* = −0.45910 + 1.01927*x*. For both models, *y* = predicted pHF or pHI urine PH and *x* = pH by pHP, respectively.

Bland-Altman plots were used to visually assess the agreement between pHP and pHF and pHI. The Bland-Altman method calculates the bias estimate, which is the mean difference between 2 methods of measurement and the 95% CI of agreement (±1.96 SD). The Bland-Altman plot demonstrated that pHF and pHI measured UpH 0.34 points greater than pHP (Figure 1), as indicated by the mean difference represented by the solid line. The 95% CI of agreement was −0.10 to 0.78 for pHF and pHI, represented by the 2 dashed lines. This is similar to the findings of Constable et al. (2019), in which the mean bias was 0.28 when comparing Multistix-10-SG to a pH meter and 0.10 when comparing Hydrion to a pH meter. In a similar study, when Multistix-SG was used, the mean bias was 0.20 compared with a pH meter (Afsahi et al., 2020). Although the mean bias was lower in the 2 previously mentioned research studies compared with the mean bias in our study, this is likely a minor difference due to the interval of measurements when determining UpH with pH paper or dipstick. To further quantify agreement between the pHP and pHF and pHI, we used the CCC. The CCC assigns values from −1 to 1, in which −1 is perfect disagreement and 1 is perfect agreement. The CCC was 0.933 (95% CI: 0.92–0.94; *P* < 0.01) for pHF and 0.926 (95% CI: 0.913–0.938; *P* < 0.01) for pHI. This indicates near-perfect agreement between pHP and the 2 sticks (pHF and pHI) used to measure UpH. The Pearson correlation coefficient, representing a linear relationship between 2 methods of measurement, also indicated near perfect agreement at 0.981 (95% CI: 0.976–0.984) for pHF and 0.978 (95% CI: 0.973–0.982) for pHI when each were individually compared with pHP. Based on these findings, either pHF or pHI would allow accurate and inexpensive measurement of UpH in dairy cows, although pHI is the least expensive option. Multistix-10-SG may be more accurate to measure UpH, likely because of the smaller interval of measurement; however, it is not readily available to producers and is more expensive than pHion balance test strips (pHI), which are easily accessible. Additionally, pHI uses a dual pad indicator on a plastic strip, which does not bleed, whereas a common issue with pH paper (e.g., Hydrion) is color bleeding once dipped in liquid.

Results of prepartum DMI and postpartum DMI, milk yield, and milk composition are in Table 1. Prepartum, the effect of UpH on DMI was determined by week due to variations in UpH weekly. There were no differences in DMI due to UpH prepartum (*P* ≥ 0.14) or postpartum DMI (*P* = 0.72). Previous research indicated that metabolic acidosis characterized by UpH ranging from 5.5 to 7.0 (Constable et al., 2009) may decrease DMI during the 3 wk before calving compared with cows not in compensated metabolic acidosis (Zimpel et al., 2018; Glosson et al., 2020). In the current study, the average UpH for the top one-third of cows (i.e., the high UpH group) was 6.42 compared with 5.44 (low) and 5.54 (medium), indicating that all cows were likely in metabolic acidosis, which was expected according to the study design. Our data indicate no advantage or disadvantage in terms of DMI to have a greater UpH while still being within the classification of induced metabolic acidosis. There was a tendency for an UpH × week interaction for milk yield (*P* = 0.09; Figure 2A). Greater milk yield at wk 1 postpartum for cows in the high UpH group may indicate a detrimental carryover effect if UpH is averaged at <5.54 (cows in the low and medium groups) and may suggest that UpH ranging from 6.0 to 7.5, deeming the diet partially acidified (Cardoso et al., 2020), may be more beneficial for milk yield. However, yields of ECM and 3.5% FCM were not affected by UpH (*P* ≥ 0.26). Additionally, milk composition content and yields were not different for cows characterized as having low, medium, or high UpH (*P* ≥ 0.12), excluding total solids. There was a tendency for a UpH × week interaction for total solids content (*P* = 0.06; Figure 2B). Greater milk total solids for cows in the low group is likely due to lesser milk yield in those cows (Fuertes et al., 1998).

**Table 1:** Least squares means and associated SEM for prepartum dry matter intake (DMI) and postpartum DMI and milk yield and composition of Holstein cows characterized by their urine pH (UpH) for 4 wk before calving

Variable^1^	Treatment^2^			SEM^3^	*P*-value^4^		
	Low	Medium	High		UpH	Week	UpH Ã— week
Prepartum^5^							
DMI, kg/d							
wk −4	13.4	12.9	12.2	0.49	0.23	—	—
wk −3	12.8	11.8	12.9	0.43	0.14	—	—
wk −2	11.5	11.4	11.8	0.47	0.83	—	—
wk −1	10.8	10.0	10.0	0.59	0.53	—	—
Postpartum^6^							
DMI, kg/d	17.0	17.8	17.6	0.77	0.75	<0.01	0.72
BHB, mmol/L	0.73	0.68	0.68	0.07	0.87	0.27	0.31
Milk yield							
Milk yield, kg/d	38.7	39.7	41.1	1.69	0.63	<0.01	0.09
ECM, kg/d	46.2	47.4	47.0	2.05	0.91	0.02	0.29
3.5% FCM, kg/d	47.6	48.6	47.9	2.21	0.95	<0.01	0.26
Milk composition							
Fat, %	4.58	4.47	4.42	0.16	0.77	<0.01	0.65
Protein, %	3.27	3.29	3.38	0.06	0.35	<0.01	0.49
Casein, %	2.67	2.71	2.75	0.05	0.53	<0.01	0.31
Fat, kg/d	1.84	1.86	1.82	0.10	0.95	0.97	0.26
Protein, kg/d	1.31	1.37	1.40	0.05	0.51	0.21	0.39
Lactose, kg/d	1.94	2.01	1.98	0.08	0.81	<0.01	0.52
Casein, kg/d	0.55	0.60	0.63	0.05	0.53	<0.01	0.33
SCC, Ã—10^3^ cells/mL	157	362	201	118	0.86	<0.01	0.58
Lactose, %	4.77	4.78	4.70	0.03	0.21	<0.01	0.63
Total solids, %	13.9	13.7	13.6	0.20	0.71	<0.01	0.06
MUN, mg/dL	12.8	12.0	11.3	0.50	0.15	0.43	0.56

1 Data were collected daily and consolidated to weekly averages for 4 wk prepartum and 4 wk postpartum.

2 Treatments consisted of UpH collected from cows weekly and classified, by terciles, as low, medium, or high.

3 Greatest value for standard error of the mean within treatment.

4 Consists of main effect of UpH, week, and the interaction of UpH Ã— week.

5 Terciles for prepartum data were determined independently by week. Terciles for wk −4 (n = 53): low (UpH â‰¤5.55; mean Â± SD: 5.36 Â± 0.15), med (UpH >5.55 and â‰¤5.78; 5.65 Â± 0.06), or high (UpH >5.78; 6.51 Â± 0.57). Terciles for wk −3 (n = 80): low (UpH â‰¤5.49; mean Â± SD: 5.35 Â± 0.11), medium (UpH >5.49 and â‰¤5.77; 5.62 Â± 0.09), or high (UpH >5.77; 6.62 Â± 0.69). Terciles for wk −2 (n = 83): low (UpH â‰¤5.37; mean Â± SD: 5.25 Â± 0.10), medium (UpH >5.37 and â‰¤5.65; 5.50 Â± 0.08), or high (UpH >5.65; 6.46 Â± 0.68). Terciles for wk −1 (n = 83): low (UpH â‰¤5.48; mean Â± SD: 5.36 Â± 0.11), medium (UpH >5.48 and â‰¤5.80; 5.66 Â± 0.09), or high (UpH >5.66; 6.48 Â± 0.54).

6 Terciles for postpartum data were determined by averaging UpH for the 4 wk before calving for each cow (n = 75) and then classified as low (UpH â‰¤5.54; mean Â± SD: 5.44 Â± 0.07), medium (UpH >5.54 and â‰¤5.90; 5.67 Â± 0.09), or high (UpH >5.90; 6.42 Â± 0.36).

**Figure 2:**
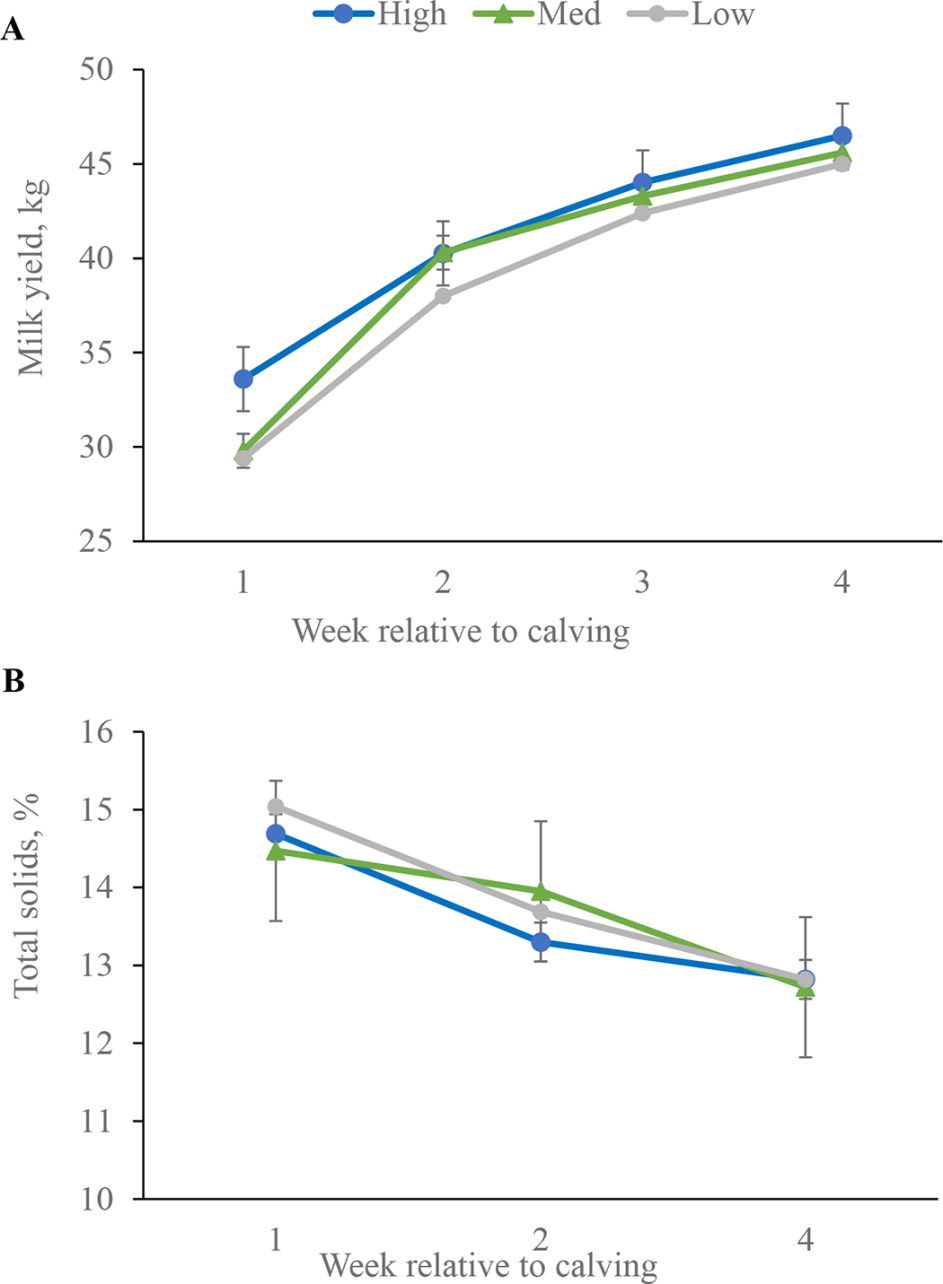
Least squares means (±SEM) for milk yield and total solids content for 4 wk postpartum by urine pH (UpH) classified as high (UpH >5.90; mean ± SD; 6.42 ± 0.36), medium (med; UpH >5.54 and ≤5.90; 5.67 ± 0.09), or low (UpH ≤5.54; 5.44 ± 0.07). Cows were consuming a negative DCAD diet of −118 mEq/kg achieved by the addition of an anionic mineral supplement. Cows (n = 75) began consuming the diet 4 wk before calving until calving. Effect of UpH for milk yield (*P* = 0.63) and milk total solids content (*P* = 0.72). Interaction of UpH × week for milk yield (*P* = 0.09) and milk total solids content (*P* = 0.06).

Further analysis of the data revealed variability in the standard deviations within the UpH terciles. For all terciles, the group characterized as high had the greatest variability, as indicated by greater standard deviations. For example, when UpH was averaged for each cow prepartum, the standard deviation for the high group was 0.36, whereas it was 0.07 for the low group and 0.09 for the medium group. This greater variability for the high UpH group may correspond to greater difficulty ensuring that the UpH of cows stays within the range indicated for this group. This is likely due to acid–base balance, in which the amount of strong acid within the UpH (as H^+^ ions) exceeded the buffering capacity (Jørgensen, 1957), resulting in a decrease in pH that was sustained for cows in the low and medium groups or those with an average UpH <5.54. An additional consideration regarding classification of metabolic acidosis based exclusively on DCAD level, which has previously been used to estimate metabolic acidosis (Lean et al., 2019), rather than UpH, is that determining the DCAD only may not be adequate and could lead to increased variability (Cardoso et al., 2020). In the current study, all cows consumed the same negative DCAD diet but UpH ranged from an average of 5.44 to 6.42, suggesting that many factors other than DCAD affect UpH. However, it should also be noted that according to Constable et al. (2019), UpH is only an adequate estimation of net acid excretion when >6.11, although UpH <6.11 does accurately predict Ca excretion of ≥4 g/d.

Based on the results obtained from this study, we conclude that Fisherbrand pH strips and pHion strips are both accurate and inexpensive methods to measure UpH. Additionally, the degree of metabolic acidosis, as characterized by UpH, does not affect prepartum or postpartum DMI. However, there may be unfavorable effects on milk yield during wk 1 postpartum if average UpH is <5.54 during the prepartum period, which may be economically detrimental to producers because of the increased cost associated with a greater anion inclusion rate needed to achieve that UpH.
